# Genome Sequence of a Circulating Human-Like Swine-Origin Influenza A Virus H3N2 Strain

**DOI:** 10.1128/genomeA.00907-13

**Published:** 2013-11-14

**Authors:** Xinsheng Li, Junya Wang, Hong-Ying Chen, Ruoqian Yan, Xiangdang Du

**Affiliations:** Department of Microbiology and Immunology, College of Husbandry and Veterinary Medicine, Henan Agricultural University, Zhengzhou, People’s Republic of Chinaa; Zhengzhou Center for Animal Disease Control & Prevention, Animal Husbandry Bureau of Henan Province, Zhengzhou, People’s Republic of Chinab

## Abstract

The full-genome sequence of A/swine/Henan/1/2010, a strain of influenza A virus isolated in central China, was determined. Phylogenetic analyses show that its eight genomic segments are human-like, and some of its segments have appeared in swine H1N2, swine H1N1, and human H1N2 influenza viruses.

## GENOME ANNOUNCEMENT

Swine influenza virus (SIV) contains eight separate negative-sense RNA segments and belongs to the *Orthomyxoviridae* family (1, 2). The influenza A viruses are divided into subtypes based on two surface glycoproteins, hemagglutinin (HA) and neuraminidase (NA). Only some influenza A virus subtypes (i.e., H1N1, H1N2, and H3N2) are currently in general circulation among humans. At the same time, viruses of the H1N1, H1N2, and H3N2 subtypes are enzootic in swine (3, 4). Swine play an important role in influenza virus infections (3, 5). Human-origin influenza A viruses can infect swine. Antigenic drift and antigenic shift may occur in the viruses over time. This will produce new virus strains that may not be recognized by antibodies to earlier influenza virus strains, or it may result in a new influenza virus that can infect humans and has an HA protein or HA and NA protein combination that has not been seen in humans for many years. This will lead to people getting the flu more than once. Therefore, understanding the evolution of swine origin influenza virus is greatly important to the prevention of influenza in the world.

In this study, strain A/swine/Henan/1/2010 (H3N2) (SW/HN/1/10) was isolated from nasal swab samples of pigs with influenza-like clinical symptoms in the Henan province of central China. PCR was performed by using the universal primers for influenza A virus (6). The sequencing results revealed that strain SW/HN/1/10 consists of eight genes, polymerase basic 2 (PB2), PB1, polymerase acidic (PA), HA, nucleoprotein (NP), NA, matrix (M), and nonstructural (NS), whose full lengths are 2,341, 2,341, 2,233, 1,728, 1,565, 1,477, 1,027, and 890 nucleotides, respectively.

Genome sequence analyses showed that there are 11 potential N-linked glycosylation sites in HA (positions 24, 38, 54, 79, 138, 142, 149, 181, 262, 301, and 499) proteins and 9 in NA (positions 61, 70, 86, 93, 146, 200, 234, 329, and 402) proteins.

Interestingly, the PB1, NP, and NA genes of SW/HN/1/10 strain are approximately 99% identical at both the nucleotide and amino acid levels to the PB1, NP, and NA genes of swine H1N2 viruses, respectively; the PA gene is approximately 99% identical at both the nucleotide and amino acid levels to the PA genes of swine H1N2 and human H1N2; and the NS gene is also 99% identical to that in the swine H1N1 and swine H1N2 viruses. This information tells us that with SW/HN/1/10 circulating in pigs, one or more genes shifting among swine H1N2, swine H1N1, swine H3N2, human H1N2, and human H3N2 viruses are leading to one or more new reassortants being produced.

### Nucleotide sequence accession numbers.

The complete genome sequence of A/swine/Henan/1/2010 (H3N2) has been deposited in GenBank under the accession no. KF541238, KF550964, KF541239, KF277766, KF541240, KF541237, KF550963, and KF550962 for segments 1 to 8, respectively.
